# Impact of natural resources and research on cancer treatment and prevention: A perspective from Cameroon

**DOI:** 10.3892/mco.2013.132

**Published:** 2013-05-27

**Authors:** BARNABAS BESSEM ORANG-OJONG, JOSE EDWARD MUNYANGAJU, MA SHANG WEI, MIAO LIN, FAN GUAN WEI, CHARLES FOUKUNANG, YAN ZHU

**Affiliations:** 1 Tianjin State Key Laboratory of Modern Chinese Medicine, Institute of Traditional Chinese Medicine and Development, Tianjin University of Traditional Chinese Medicine, Tianjin 300139, P.R. China;; 2 Department of Pharmacy and Traditional Pharmacopoeia Development, University of Yaoundé 1, Yaoundé and Faculty of Medicine University of Bamenda, Yaoundé 00237, Cameroon

**Keywords:** cancer, anticancer therapy, medicinal plants, treatment, prevention, natural resources

## Abstract

Cancer is a significant public health concern and treatment poses a problem and is frequently unsuccessful. As such, continuous efforts in the search for new agents and therapies to improve survival are required. A considerable number of plant extracts and isolated compounds possess significant anti-proliferative or pro-apoptotic effects. The majority of biologically active compounds belong to terpenoids, phenolics and alkaloids. Terpenoid plants such as *Hypericum lanceolatum* and a few alkaloid plants have been found to be potent anti-parasitic agents but have exhibited poor antimicrobial effects. The screening of medicinal plants for anticancer drugs has provided modern medicine with effective cytotoxic pharmaceuticals. Numerous medicinal plants have traditionally been used for the treatment of various ailments. However, a number of these medicinal plants have not been standardized and their mechanisms of actions are generally unknown. Active drug discovery research using local medicinal plants is ongoing. Some of these plant-derived compounds, including 3,39-dimethoxy-49-*O*-β-d-xylopyronosylellagic acid, have been tested for their potential use as anticancer agents. This review discussed the scope and possibility of natural products as anticancer remedy.

## Contents

1. Introduction
2. Background
3. Rationale
4. Methods for conducting information search
5. Government agenda
6. Cameroon cancer statistics
7. Quality of care to cancer patients in Cameroon
8. Government interventions
9. National cancer control
10. Community interventions
11. Current status of radiotherapy and chemotherapy services in Africa and Cameroon
12. Limitations in resources: Anticancer drugs
13. Limitations of resources: Radiotherapy
14. Ethno-search for cancer therapy in Cameroon
15. Clinical cancer study efforts
16. Knowledge gaps
17. Conclusion

## 1. Introduction

Cancer is considered the leading cause of death in developed countries and the second leading cause of death in developing countries (1–5).The burden of cancer is notably heavier in developing countries due to factors including age and lifestyle choices such as smoking, physical inactivity, and consumption of genetically modified foods that may lead to cancer (6). Approximately 12.7 million cancer cases and 7.6 million cancer deaths are estimated to have occurred in 2008 worldwide, with 56% of the cases and 64% of the deaths in the economically developing world (7,8). Deaths from cancer worldwide are projected to continue rising, with an estimated 13.1 million deaths expected in 2030 (9,10).

In 2008, 715,000 new cases of cancer and 542,000 new cancer deaths were recorded in Africa, rendering cancer an emerging public health concern (12). These numbers are likely to double within the next 20 years for the reasons mentioned above (1). Furthermore, an increase in the frequency of diagnosis of various types of cancer such as lung, female breast and prostate cancers has been observed due to lifestyle changes and detection practices associated with urbanization and economic development (7,8). In Cameroon, cancer accounts for 3% of deaths from all ages annually (1–4) and the cancer risk for both males and females prior to the age of 75 is 8.7% (Table I). Data concerning cancer statistics in Cameroon are generally lacking at national and institutional levels. Therefore, the aim of this review was to assess some cases from the perspective of Cameroon as a developing or low-income country.

Our knowledge regarding cancer prevention and control has increased, yet the number of cancer cases is on the increase. Should this trend continue, 16.5 million new cases of cancer are expected to be diagnosed in 2020 (1–4) with an approximate 10 million deaths expected, two-thirds of which are likely to occur in developing nations (14). Although obtaining government subsidies is crucial for the diagnosis and treatment of cancer patients in Cameroon, cancer drugs remain both expensive and scarce. Through the National Cancer Control Programs, the state of Cameroon together with partners such as Sanofi Aventis is attempting to ensure accessibility to drugs in a cost-effective manner (15).

Cameroon has recently followed the global trend of directing attention towards the search for alternative treatments and the use of active herbal compounds for the treatment of cancer and other diseases. In Cameroon, terpenoid plants such as *Hypericum lanceolatum*(16) have been identified as extremely potent anti-parasitic agents but have demonstrated poor antimicrobial effects (17–19). This review discusses the scope and potential impact of natural products from Cameroon as anticancer remedy.

## 2. Background

Cameroon is a central African country whose natural environment covers four bio-ecological zones from south to north: the equatorial forest, the Guinea savannah, the Sahel savannah and the encroaching desertification in the far north. Commonly referred to as Africa in miniature, Cameroon has an estimated 250 ethno-linguistic groups from five regional-cultural groups spread across the various ecological zones: the Semi-Bantus (western highlanders) including the Bamileke, Bamoun and numerous smaller entities in the North-West situated in the Guinea Savannah, who constitute 38% of the population; the Bantus, coastal tropical forest peoples, including the Bassa, Douala and numerous smaller entities in the South-West, who constitute 12% of the population; the southern tropical forest peoples, including the Ewondo, Bulu, and Fang (all Beti subgroups), Maka and Pygmies (known as Bakas), who constitute 18% of population; the predominantly Islamic peoples of the northern regions (the Sahel) and central highlands, including the Fulani, who constitute 18%; and the Hamites and Semites peoples of the Sahel and central highlands, who constitute 14% of the population (20–22). This ethnic and cultural diversity impacts on the perception, treatment-seeking behavior and care for cancer and related illnesses.

**Figure 1:**
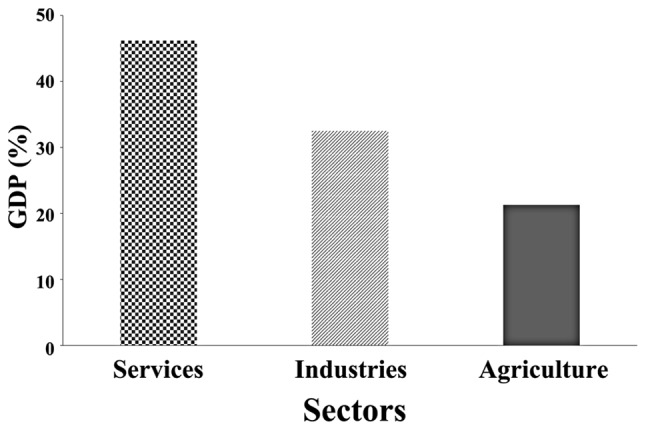
Cameroon gross domestic product (GDP) by sector. Services contribute 46.20% to the GDP, industries 32.5% and agriculture 21.3% (23).

According to the 2008 statistics of the WHO, the population of Cameroon is 19.088 million, and as with the majority of African countries, has a low-income economy (1–4). Over the past 5 years the average gross domestic product (GDP) growth of the country has been 3.5%, below the originally estimated 5% and drawn mainly from three sectors (Fig. 1) (23). Recent figures have revealed that, between 2000 and 2009 the country experienced economic growth at an average annual rate of 3% (24). An increase in the growth of trade with a valued import and export of 56% has also been observed (24). As such, there are ongoing social and economic changes resulting in increased urbanization with potentially negative consequences that could affect health in the short- or long-term.

However, there is great disparity in the distribution of wealth generated by economic growth, with 32.8% of the Cameroonian population living below the poverty line (<US$1/day) (1–4). Thus, the poverty-stricken primary victims of cancer and other diseases have limited access to health services, due to the high cost of diagnosis and treatment (∼2.1 million francs) (25,26). The cost of health services is beyond the means of an average Cameroonian according to officials of the National Cancer Control Committee (NCCC) (23,25,27). As a consequence of the aforementioned socio-economic changes, Cameroon may now be experiencing a double burden of infectious and chronic non-communicable diseases (NCDs). The burden of infectious diseases largely driven by malaria, HIV/AIDS and tuberculosis is already weighing heavily on the economy. However, an increase in the burden of NCDs, exhibiting elements of a health transition, in which a combination of acute and chronic diseases coexist in the same population and compete for limited resources has been a critical issue (13,27).

## 3. Rationale

In their global statistic paper, Jemal *et al* (7,8) concluded that a ‘significant proportion of the worldwide burden of cancer could be prevented through the application of existing cancer control knowledge, and by implementing programs for tobacco control, vaccination (for liver and cervical cancers) and early detection and treatment, as well as public health campaigns promoting physical activity and healthier dietary patterns’ and that ‘implementing and sustaining such actions requires concerted efforts among private and government public health agencies and the pharmaceutical industry, as well as individual and government donors’. In this study, we assess the efforts made by both the government and private sector in the fight against cancer in Cameroon in the areas of research, treatment and prevention. Emphasis is laid on research activities in the field of cancer, particularly in the area of anticancer research with regard to herbal sources and alternative treatments (28–30). Key questions to be addressed include: i) whether the cause of the increased incidence of cancers can be identified; ii) which interventions are effective and which interventions fail to improve the quality of care of the cancer care receiver; iii) how to utilize the local and unique natural resources to reduce the cost of cancer treatment. These questions may be addressed by examining the literature available on the prospect of cancer research in Cameroon with a focus on the impact of natural resources and research on cancer treatment and prevention.

## 4. Methods for conducting information search

The literature was reviewed and a search was conducted using keywords including cancer, Cameroon, epidemiology, management, research, herbs, as well as a combination of these in different permutations. A number of studies on cancer and associated diseases in Cameroon were reviewed for this study (1–4,15,18–20). The review focused on published and gray literature on anticancer activities of the government of Cameroon, Cameroonians and all stakeholders involved in the domain of cancer. Cameroonian investigators as well as research organizations on cancer were also contacted for additional information and gray literature. The search was also carried out in the TUTCM e-library. Search methods were manual and electronic. Databases and search engines including Google, Yahoo, Hinari and PubMed were searched to access other relevant publications. International organizations such as WHO, UNICEF, GTZ, IFORD and the UNDP were also contacted for possible information through their websites. The keywords used in the search were based on cancer and Cameroon. Several authorities were consulted through personal communication to complement the review of the literature. Any documents consulted were both in English and French, the two official languages of Cameroon.

## 5. Government agenda

In its growth and strategy manuscript of 2010, the government of Cameroon established specific aims constituting a reference framework for: i) reducing poverty to a socially acceptable level; ii) reaching middle-income country status; iii) becoming a newly industrialized country; iv) consolidating the democratic process and strengthening national unity (23). To attain this general vision, the specific objectives of the strategy involved: i) increasing growth to an annual average of 5.5% over the 2010–2020 period; ii) reducing underemployment from 75.8 to below 50% in 2020, with the creation of numerous formal jobs over this 10-year period; and iii) reducing the monetary poverty rate from 39.9% in 2007 to 28.7% in 2020. To achieve these objectives, the government intends to implement in a coherent and integrated manner: i) a growth strategy, ii) an employment strategy, and iii) a strategy to improve governance and the central government’s strategic management (23).

To attain specific objectives, for example, objective ii), the government launched a mass recruitment of 25,000 youths from all disciplines into the civil service in 2011. Actions such as these are paramount in ensuring that chronic diseases such as cancer, currently faced with expensive health care services, receive more attention in economic terms.

## 6. Cameroon cancer statistics

GLOBOCAN statistics of Cameroon showed that the age-standardized rate of cancer deaths is 73.1/100,000 persons/year and the risk of succumbing to any type of cancer prior to the age of 75 was 11% (7–10,31). The most common cancer types are breast, uterine cervix, liver, non-Hodgkin’s lymphoma and prostate cancer. Prostate cancer is the most common malignancy in men, with an age-standardized incidence rate of 19.2 and a mortality rate of 15.2/100,000 persons/year. In women, breast and cervical cancers are the most prevalent tumors, with breast cancer recording the age-standardized incidence rate of 27.9 and a mortality rate of 16.6/100,000 persons/year. For cervical cancer, the age-standardized incidence rate is 24 and mortality 19/100,000 persons/year (1–4,7,8). Other less frequent female genital cancers in order of decreasing frequency include ovarian, endometrial and vulva. These cancers, often diagnosed at advanced stages, result in the belief that cancer is incurable and therefore lethal. Efforts have been under way to reverse these statistics in the area of early diagnosis, treatment and prevention in recent years. Several studies support the existence of screening programs for cervical, breast and prostate cancers in Cameroon (32–34). However, the impact of the activities of these programs on prevalence, incidence and death rates have yet to be determined. Therefore, whether these programs have contributed to reducing cancer-related deaths over time is unclear. As the NCCC registry does not extend to the national territory, there is no national population-based cancer registry, although one is available for the capital city, Yaoundé (35). Due to the attitudes and beliefs of the population regarding cancer, a national cancer screening program is organized periodically, although only in the regions of Yaoundé and Douala. As such, the rural population, which is most affected, is not included.

**Figure 2:**
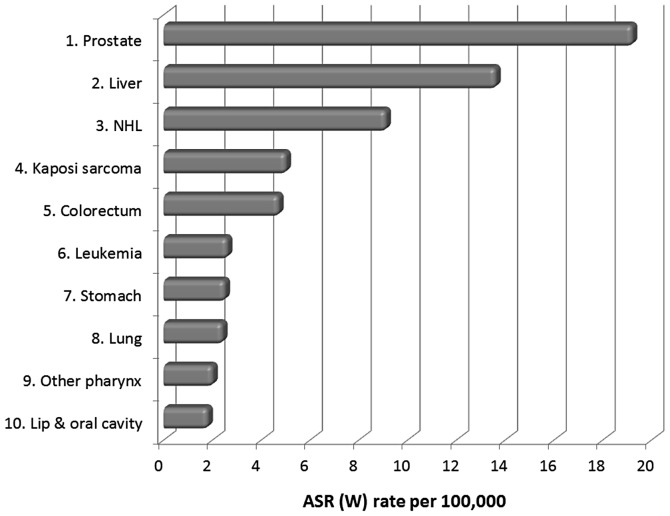
Ten most common cancers among men in Cameroon, including prostate cancer, liver cancer, non-Hodgkin’s lymphoma (NHL), Kaposi sarcoma, colorectal cancer, leukemia, stomach cancer, lung cancer, other pharynx and lip and oral cavity. Age-standardized rate (ASR) is the potential rate of a population if a standard age structure was available. This rate is the number of new cancer cases/100,000 populations/year (10,14).

**Figure 3:**
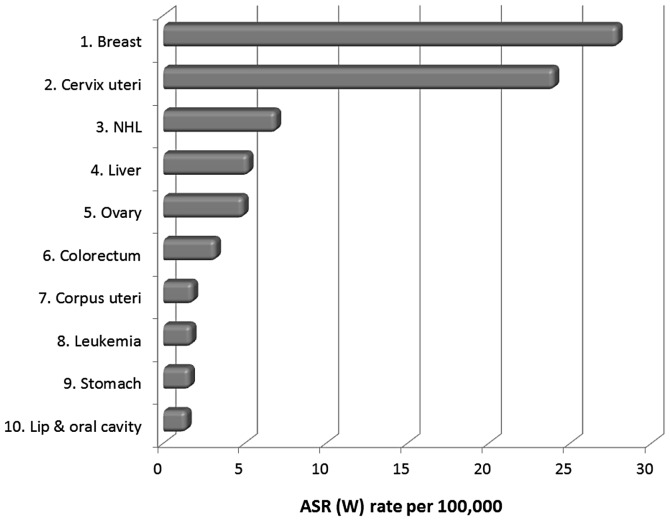
Ten most common cancers among women in Cameroon including breast cancer, cervical cancer, non-Hodgkin’s lymphoma (NHL), liver, ovarian, colorectal and uterine cancer, leukemia, stomach cancer and lip and oral cavity (10,14).

**Figure 4:**
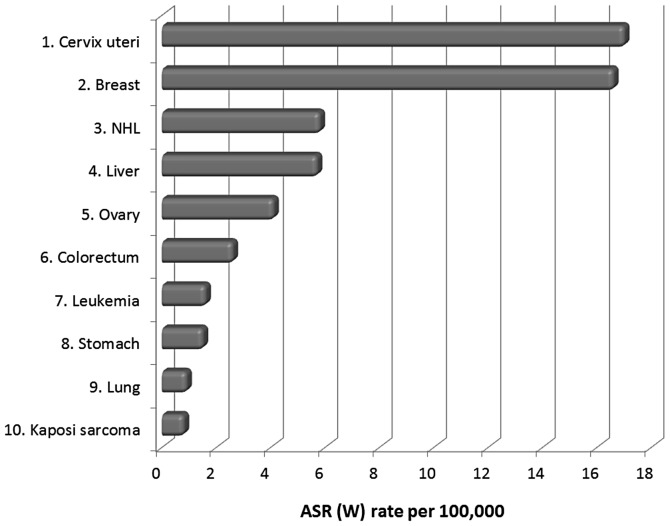
Ten leading cancer deaths among women in Cameroon including cervical cancer, breast cancer, non-Hodgkin’s lymphoma (NHL), liver, ovarian and colorectal cancer, leukemia, stomach cancer, lung and Kaposi sarcoma (10,14).

**Figure 5:**
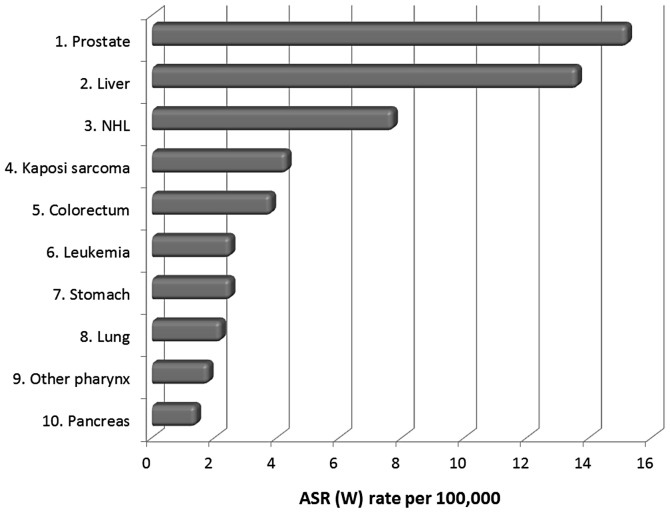
Ten leading cancer deaths among men in Cameroon including prostate, non-Hodgkin’s lymphoma (NHL), Kaposi sarcoma, colorectal cancer, leukemia, stomach cancer, lung cancer, other pharynx and pancreas. (10,14).

The international agency for research on cancer (IARC) in 2008, estimated the top 10 population-based age-standardized incidences of cancers for both men and women in Cameroon (Figs. 2–5). These data indicate that the rate of cancer prevalence and related deaths is critical. Prostate cancer is the most common cancer among men in Cameroon (Fig. 2), followed by liver cancer, non-Hodgkin’s lymphoma (NHL), Kaposi sarcoma, colorectal cancer, leukemia, stomach and lung cancer. In women, breast cancer is the most common type of cancer (Fig. 3), followed by cervical cancer, non-Hodgkin’s lymphoma (NHL), liver, ovarian, colorectal and uterine cancer, leukemia, and stomach cancer. The leading cause of cancer-related deaths among women in Cameroon is cervical cancer (Fig. 4), followed by breast cancer, non-Hodgkin’s lymphoma (NHL), liver, ovarian and colorectal cancer, leukemia, and stomach cancer, whereas in men, it is prostate cancer followed by other cancer types (Fig. 5).

Of note is evidence of male breast cancer in Cameroon. In a study conducted by Ndom *et al* (15) a male-to-female breast cancer ratio of 3.7% was noted. Therefore, male breast cancer should be considered in the fight against cancer.

## 7. Quality of care to cancer patients in Cameroon

Cancer surveillance in Cameroon is scarce and where it occurs, it is usually not well-organized, while the records for cancer-related deaths are inaccurate as the majority of cancer-related deaths are not reported or recorded due to limited services available in most areas, with the exception of Yaoundé and Douala (36,37). Patients usually present at hospital with an advanced stage of the disease (15,32,35) due to various factors including ignorance, local beliefs and poverty, although the reasons for delayed diagnosis and treatment remain to be elucidated in qualitative and quantitative investigations. Delay in hospital visitations has also been compounded by traditional healers, medical and paramedical staff, who for financial or other reasons, treat cancer patients without appropriate skills. This is exacerbated by the fact that cancer treatment centers are centralized in Yaoundé and Douala only, allowing patients to seek alternative treatement and care. This situation can be improved by identification and training of traditional healers whose use of herbs has provided evidence of tumor regression. The anti-proliferative activities of their decoctions should be tested and better guided in their practice (32).

Inadequacy of equipment limits the practice of cancer case management and treatment in most African countries (12,38). In Cameroon, oncology is not a popular medical specialty among young medical officers and nurses, which aggravates the shortage of care providers to these patients. Additionally, despite the National Committee’s successful efforts to secure drugs at a reduced cost, drugs used for chemotherapy are expensive. Cancer control in national health development programs has been promoted, however, adequate funding to achieve these goals is critical.

## 8. Government interventions

The government of Cameroon has ratified the WHO’s Framework Convention on Tobacco Control (FCTC), but it remains largely non-compliant to any existing FCTC measures (39). A number of tobacco control measures exist in Cameroon, including some limited smoke-free legislations, which, however, are frequently not implemented. Drope refers to this lack of implementation as ‘the politics of smoke-free policy’ (40). Additionally, the national tobacco control program keeps no data regarding the trends in tobacco consumption and consumer behaviors in public places, and the progress of this program is hardly evaluated (41).

Health warnings on cigarette packs, such as ‘cigarette smoking is dangerous to your health’, and advertisements against smoking are recent events in Cameroon as the national tobacco control and taxation bill did not include all aspects of the FCTC. Thus, there is a need for more effective tobacco control programs, as well as to enforce the legislation that prohibits smoking in all public places, address the issue of free advertising by the tobacco industry, increase public awareness of the health hazards associated with cigarette smoking and other tobacco products, as well as restrict the access of young persons to cigarettes (39).

This campaign has faced great setbacks partly due to cigarette smoking being a lifestyle choice for most uniformed services in Cameroon, particularly the military and gendarmerie. Cameroon has only recently attempted to actively implement smoke-free regulations and directives, such as the banning of smoking in public buildings by the ministry of Public Health. Recent studies have reported an informal ban on smoking on public transportation services (6). In late 2009, a team of tobacco control advocates, funded by the ATSA initiative, initiated a program to implement smoke-free policies in Mfoundi beyond just government buildings to include other public (e.g., hospitals, educational institutions, healthcare facilities and tourist establishments) and private environments (e.g., workplaces) (42,43). In this ongoing effort, the advocacy team continues to engage civil society organizations, enforcement officials and the local authorities as key partners.

At present, no national food policy exists in Cameroon. A few legislations guiding the registration of food products in Cameroon are applicable with a mandate on food products to demarcate the following information: food labeling, nutritional value of foods, microbial content and additives clearly evident on packaging (44). However, 32% of the Cameroonian population is illiterate (3,4) and a great proportion of the general population is illiterate and not likely to read the packaging information on these products.

## 9. National cancer control

In a telephone interview to an undisclosed source, Dr Anderson Doh of the Faculty of Medicine and Biomedical Sciences of Yaoundé addressed key questions pertaining to cancer control in Cameroon. From his responses, a national committee for the control of cancer was set up on October 24, 1990 whose activities were limited and only consisted of periodic screening campaigns (2 or 3 times/year) for cancers of the cervix, breast and prostate. Occasional screening campaigns were also organized by a few specialists. These activities were, however, not coordinated.

The NCCC was reorganized by ministerial decision N°0153/MSP/CAB of 13 January 2002, and charged with six general objectives and nine specific objectives. Priority objectives involved the prevention of cancer, diagnosis and treatment of cancer cases, collection of data, undertaking of research and mobilization of resources. In 2004, it published a comprehensive plan including all the main components. This plan cannot be fully implemented, however, due to lack of funds. After implementing its 2006/2020 strategic plan, the NCCC was revised.

Anti-mitotic drugs are provided at <40% the cost in private pharmacies with funding from the Ministry of Health, especially funds from the ‘Heavily Indebted Poor Countries (HIPC) Initiative’ (25). Individuals take care of their patients. Since 80% of cancer patients present at a late stage of the disease, palliative care is a significant component of the NCCC. On 6th April 2006, the Ministry of Health signed a partnership agreement with the International Network for Cancer Treatment and Research (INCTR) with the objective of initiating a palliative care program. As from 2006, analgesics used for pain treatment were subsidized by the ministry of health. A national vaccination for hepatitis B as a preventive measure was initiated in 2005 to help limit the development of liver cirrhosis and viral-induced liver cancer in Cameroonians.

The overall objective of the NCCC is to reduce cancer-related morbidity and mortality in Cameroon and to create a cancer registry, albeit the data currently available for this registry are drawn from the district, regional and referral hospitals only. Information from the rural areas is therefore lacking, particularly as the effect of the NCCC is evident only in Yaoundé and Douala at major hospitals.

Notably, in 2004, the government signed a convention with the pharmaceutical company Sanofi Aventis, which resulted in the reduction of cost of the company’s most effective cancer drug (taxotere) to ∼70%. The drug according to company officials had been previously sold at 480,000 Francs, while following the convention the cost was set at 170,000 Francs. As part of activities to mark the national cancer prevention week in Cameroon a series of surgical operations were carried out by a team of American and Cameroonian specialists. Specialized cancer treatment centers are available where early stages of cancer can be diagnosed and treated. These centers also provide palliative and psychological care to cancer patients. Considering that some cancer diagnoses are extremely expensive, the NCCC has developed more cost-effective but highly efficient procedures to diagnose certain types of cancer. The NCCC has produced and distributed numerous detailed documents on cancer and trained a large number of health personnel on cancer-related issues. Well-equipped mobile diagnosis and treatment teams proceed from Yaoundé to towns and villages around the country to carry out awareness campaigns, as well as screening and treatment of cancer cases on a volunteer basis.

## 10. Community interventions

Nationwide campaigns to promote physical activity, good nutrition, and tobacco control do not exist. Instead, sporadic activities are often organized around particular events such as the national cancer week that are usually ceremonial and limited to health administrative regions such as Yaoundé and Douala. These health promotion activities used both conventional (mass media, health facilities and distribution of health education materials) and non-conventional (meetings in market places, in churches/mosques and health education activities in schools) methods to promote good health. The Cameroon Baptist Convention Health Board runs a cervical prevention and Women’s Health Programme (WHP) in the private confessional sector. Evaluation studies on the impact of these interventions on health outcome are lacking as do cost effectiveness analyses of these interventions. Therefore, which intervention is most effective remains to be determined.

## 11. Current status of radiotherapy and chemotherapy services in Africa and Cameroon

**Figure 6:**
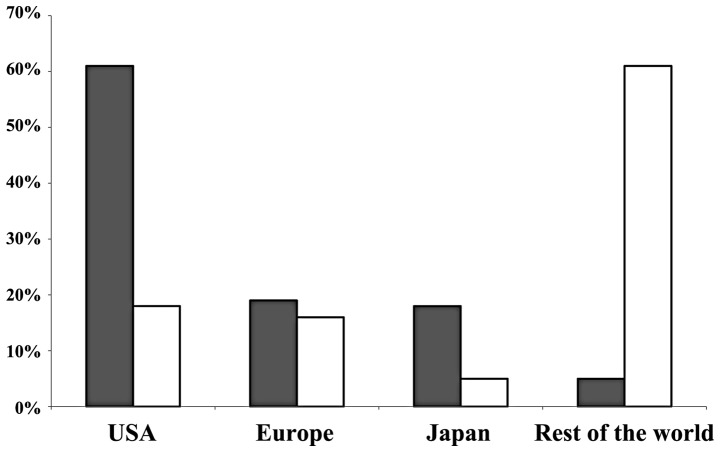
Global inequity in cancer care. Cancer drug sales (grey bars) versus cancer burden (white bars) from three regions of the world and the rest of the world (72).

Resources for the treatment of Cancer in Cameroon as with many developing countries are very limited. Among develo ping countries, the five-year cancer survival is lower in African nations compared to developing countries in Asia and South America (45). Poor cancer survival is a strong indication that additional investment is needed to improve the quality and accessibility of health services, disease awareness, and studies into cancer prevention, early detection, and treatment (45,46). The INCTR reported the global anticancer drug sales (Fig. 6).

## 12. Limitations in resources: Anticancer drugs

Fig. 6 shows the percentage of anticancer drug sales (grey bars) and the percentage of cancer burden (white bars). The USA accounts for 61% of the anticancer drug sales in the world, but only has 18% of the world’s cancer burden. By contrast, the group, the ‘rest of the world’, which largely comprises low- and middle-income countries, indicates clear global inequity in cancer care (10,14). These countries account for 61% of the world’s burden of cancer (47), but only 5% of anticancer drug sales. In Cameroon, a 20-year descriptive retrospective study carried out between March, 1989 and March, 2009, based on the registry and medical records of patients attending the Radiation Therapy Unit of the Yaoundé General Hospital by Kemfang *et al* (36), revealed that 57.55% of patients received neoadjuvant chemotherapy and 27.32% adjuvant chemotherapy, a total of 84.87% of patients receiving chemotherapy. They also observed that the benefit from endocrine therapy was substantial, in that study, in the absence of hormone receptor status: tamoxifen was proposed to all patients, with the exception of lymphoma cases, and was used by 84.11% of patients (36,38). However, 15.89% of patients were not able to afford tamoxifen. Aromatase inhibitors or activators (anastrozole, letrozole, and exemestane) that have demonstrated better efficacy than tamoxifen, could also not be utilized due to their lack of availability or high cost (36).

Although the results appear promising, it is crucial to note that these were data derived from only the radiation unit of a single hospital of breast cancer patients over 20 years from a population with annual cases of cancer of >11,700 patients (1–4).

When compared with developed and middle-income countries, it is evident that there is a need to intensify search for anticancer lead compounds from local Cameroonian herbs and the development of alternative therapy for cancer case management in Cameroon. Based on the evidence above, the search for hormonal therapy from natural sources would be a possible relief for the Cameroonian cancer patient and care giver. For example, whether the potential use of phytoestrogens derived from the Cameroonian natural flora offers a cheaper alternative to the current synthetic and expensive hormonal therapies should be investigated. In Cameroon, traditional medicine is important in health care, with 80% of people being treated for diseases such as malaria. Traditional medicine is widespread in rural areas where people do not always depend on ‘Western’ medicine (48). Thus, it is necessary to determine which interventions of the Cameroonian traditional medical systems require strengthening to be of benefit in cancer treatment.

## 13. Limitations of resources: Radiotherapy

By 2004, there were ∼2,500 radiotherapy centers and 3,700 machines available for cancer treatment in the developing world. Additionally, although there was a pressing need for radiotherapy for ∼3 million patients in the developing world, the number of centers and machines available in those countries were sufficient for only 1.85 million patients. By contrast, countries including Cameroon had only one machine available for over 19,000,000 patients in contrast to one machine for 250,000 patients in high-income countries (11,46,49). Over 20 countries, mostly in sub-Saharan Africa had no radiotherapy machines according to the International Atomic Energy Agency standards. To compound the problem, many existing machines were not being used due to a lack of maintenance of the equipment, expired sources of cobalt, or a lack of radiotherapists or physicists (26). Little change has been noted in this situation in the last 8 years for many African countries.

In a comparison between high-income countries (HIC) and low- and middle-income countries (LMICs), it was observed that between 50 and 60% of patients in HIC diagnosed with cancer were administered radiotherapy at some point during their treatment, while for patients living in LMICs, radiotherapy remains an unattainable treatment option, with only 25% of radiotherapy patients in LMICs having access to the radiotherapy treatment. There is therefore a need to increase the survival rate (11). While in some HIC, external radiotherapy only accounts for 5% of the total cost of cancer care, for many LMICs the infrastructure for and capital costs of initiating radiotherapy are extremely high, sometimes reaching >$4 million/U (26,11). When auxiliary costs such as training and maintenance are added, the total price is vastly increased (26).

Between 1998 and 2002, Cameroon had only two mega-voltage machines available for the estimated 14.5 million people, while 12,949 cases of cancer were reported (26). Between 1989 and 2009, the radiotherapy unit of Yaoundé General Hospital noted a gradual increase in the number of patients receiving radiotherapy from 3 patients in 1989 to 73 patients in 2009, suggesting a mean number of 25 patients/year out of the 531 registered cases (36). In their study, Kemfang *et al* (36) reported that 98.80% of patients were treated with radiotherapy delivered by the cobalt unit following surgery. This study reveals the great demand for these services, however, it is beyond the means of the average Cameroonian due to the high costs involved.

## 14. Ethno-search for cancer therapy in Cameroon

The screening of medicinal plants used as anticancer drugs has provided modern medicine with effective cytotoxic pharmaceuticals (50). The US for example gets most of its anticancer drugs from natural sources such as herbs (51,29). In the last decades, scientists have been exploiting the biodiversity of pathways exhibited by the flora world to unveil lead compounds that are of great value to drug development and the field of anticancer drug research is likely to benefit from these advancements in herbal medicines. Voss *et al* (52) had earlier reported the development of anticancer drugs vinblastine and vincristine from *Catharanthus roseus*. Hostettman *et al* (30) were able to show that a large number of plant extracts from plant families including Guittiferae, Rubiaceae, Apocynaceae, Euphorbiaceae and Solanaceae demonstrated great potential for anticancer activities both *in vitro* and *in vivo*(30,53). These plants are located in an exceptionally rich biodiversity, and few scientific studies have been carried out in Africa in general and Cameroon in particular, on the anti-proliferative or pro-apoptotic effects of medicinal plants. However, some studies based on Cameroonian plants are presented in the subsequent paragraphs.

In the search for anticancer drugs from herbal extracts, a few of the studies available from Cameroonian investigators are cancer-based. For example, in 2011, Kuete *et al* (54) found that the compounds xanthone V1 and 2-acetylfuro-1,4-naphthoquinone of Cameroon herbal origin showed 65.8 and 59.6%, respectively, inhibition of the growth of blood capillaries on the chorioallantoic membrane of quail eggs in an anti-angiogenic assay. Following treatment with 2-fold IC_50_ and after 72 h, the two compounds induced cell cycle arrest in the S phase, and significant apoptosis in CCRF-CEM leukemia cells. Caspase-3/7 was activated by xanthone V1. The overall results of that study provided evidence for the cytotoxicity of compounds xanthone V1 and 2-acetylfuro-1,4-naphthoqui-none, supporting their potential use in cancer therapy (54,55).

Kuete and Efferth (55) recently screened 5 Cameroonian medicinal plants widely used in cancer treatment, (*Sida acuta, Sida cordifolia,Sida rhombilifolia, Urena lobata* and *Viscum album*), for their cytoxicity against Hep G2 hepatocarcinoma cells. The results showed that these plants exhibited relatively moderate anti-proliferative effects (56). However, pronounced tumor-reducing effects were observed when extracts from the roots and leaves of *Bersama engleriana* were used on Crown Gall tumor formation (18). An IC_50_ of 27.16 *μ*g/ml was reported for *Antiaris Africana* in DU-145 prostate cancer cells, while even better activity (IC_50_ of 13.84 *μ*g/ml) was recorded in Hep G2 hepatocarcinoma cells (19). One of the most active compounds isolated from *Antiaris Africana*, 3,39-dimethoxy-49-*O*-β-d-xylopyronosylellagic acid exhibited considerable anti-proliferative activities towards Hep G2 (IC_50_ of 3.84 *μ*g/ml) and DU-145 (IC_50_ of 6.24 *μ*g/ml) cells (19). *Newbouldia laevis* (Bignoniaceae) the main constituent of a Cameroonian medicinal plant was found to be extremely active against DU-145 cells with an IC_50_ of 64.59 nM (57). Wighteone and alpinumisoflavone isolated from *Erythrina indica* (Leguminosae) were reported to be cytotoxic (effective doses of 0.78 and 4.13 *μ*g/ml, respectively) when tested against KB nasopharyngeal cancer cells (58). Globulixanthones A and B isolated for the first time in the Cameroonian medicinal plant, *Symphonia globulifera* L. f. (Clusiaceae), showed good anti-proliferative activities against human KB cells, with IC_50_ values of 2.15 and 1.78 *μ*g/ml, respectively (59). Compound values of 2.0 and 6.6 *μ*g/ml, respectively, for dihydrochelerythrine and 6-acetonyldihydrochelerythrine against *L. donovani* were noted (60).

The south-west regional pharmacopoeia developed by Jiofack *et al* (61,62) from a full-length ethno-botanical survey of 289 plant species belonging to 89 families, did not indicate anticancer components. This is mainly due to the fact that scientific methods of phytochemical extraction, isolation and characterization were not employed, nor were the subsequent scientific models for anticancer activities of the herbs tested. This would be a misrepresentation of the medicinal potential of Cameroonian herbs from the South-West and littoral regions ecological zone. Studies of this nature would present more useful data if scientific proof of the claims of the therapeutic indications of the herbalist were tested and standardized. However, despite the skills offered, lack of resources would not allow the Cameroonian ethno-scientist to explore his potential nor that of the rich ecological flora.

A similar study was conducted in the Lebialem division by Focho *et al* (63), however, results pertaining to the use of anticancer herbs in that region were not recorded. Reasons for the lack of research data involve, not only policy problems, but also the research methodology for evaluating traditional medicine likely due to lack of equipment and funds. Literature and data on the studies of traditional medicine in various ethno-research laboratories in Cameroon are available. However, their validity remains to be confirmed as most literature is based on the findings of traditional practitioners and are not scientifically viable (64). Thus, there is a need for validation and standardization of phytomedicines and traditional medical practices in order that this sector be incorporated within the health care system of Cameroon (65). As the characteristics and applications of traditional medicine differ from those of western medicine, the manner in which traditional medicine is evaluated as well as the type of academic research approaches and methods that may be used to evaluate the safety and efficacy of traditional medicine are new challenges that have emerged in recent years (65,67). Together with an increased interest in medicine is an increased interest in the safety aspects of the practice of herbal medicine (64,65,67). The private sector, which is involved in the business of herbal drugs, should take responsibility and ensure the safety and efficacy of these herbal drugs. Stringent government policies regulating the promotion of herbal medicines and products in Cameroon via advertising should be implemented, while traditional practitioners should act in a responsible manner (64).

## 15. Clinical cancer study efforts

In a descriptive retrospective study based on the registry and medical records of patients in the Gynecology and Obstetrics unit, Yaoundé General Hospital, Kemfang *et al* (36) reinforced the position occupied by late presentation and advanced stage of diagnosis of breast cancer profile in developing countries. During the study period, 531 breast cancer patients were recorded of which 0.75% were male. A total of 66.1% of the patients were <50 years old and 31.9% <40. Additioanlly, 95.34% of patients detected their cancers themselves and delayed seeking treatment at hospital for ∼1,035 months. Up to 54.94% of patients had used traditional medicine prior to medical evaluation. In another study, Ndom *et al* (15) looked into male breast cancer showing a male-to-female breast cancer ratio of 3.7%. They likewise confirmed the fact that the majority of patients (17 patients, 80%) had advanced stage at diagnosis before consulting a doctor (15).

An important knowledge gap was identified by McCarey *et al* (68) among health workers in Cameroon. Cervical cancer and prevention by screening showed several gaps and important misconceptions regarding screening methods. Most participants were aware that cervical cancer is a major public health concern (86%), were able to identify the most important etiological factors (58%) and believed that screening may prevent cervical cancer (90%) and may be performed by Pap test (84%). However, less than half were considered for VIA or HPV screening tests (38 and 47%, respectively). Thus, knowledge pertaining to cancer etiology and screening was lowest among nurses/midwives (68). However, more data are required for this finding. It would therefore be profitable if an emphasis on cancer (cervical, breast and uterine) is placed on nurse training programs as part of reproductive health. Angwafo *et al* (69) have also reported the results of prostatic biopsies from an ongoing national prostate cancer survey in Cameroon. A total of 111 men in Dibombari, aged ≥40 years, were recruited for a medical interview, PSA assay and digital rectal examination (DRE). Biopsies were performed on 24 subjects who had either suspicious prostates on DRE and/or PSA ≥4 ng/ml. Six men (5.4%) were shown to have cancer, another 6 had low-grade prostatic intraepithelial neoplasia (PIN), and another 2 (1.8%) had high-grade PIN. Sow *et al* (70) reported on urogenital malignancies in the Yaoundé Central Hospital between 1987 and 2005, identifying a total of 520 cases of prostate cancer out of 2,371 benign and malignant tumors (including over 1,000 cases of BPH). Those results indicate that prostate cancer is the most dominant malignant urogenital tumor observed in Cameroonian males.

## 16. Knowledge gaps

Gaps in knowledge of how traditional knowledge systems of health function in Cameroon, as well as their limitations and prospects following the WHO traditional medicines strategies remain (70). Successful experiences and approaches on conservation and sustainable use of medicinal plants are rare. With regard to research aspects pertaining to medicinal plants, traditional medicine and local communities, a number of questions remain to be addressed, including which traditional decoctions are effective and by what mechanism; which of the decoctions have been tested in its natural composition and how; how traditional medicinal knowledge, practices and preparations should be validated at the community levels where its use is optimal, as well as by what scientific methods and facilities; the standardization of Cameroonian traditional medicines at local and traditional healer levels in the community, and what methods and facilities should be utilized; determining the best functional conditions for indigenous medical practices; identifying the traditional medicine care receivers in Cameroon and the conditions under which they seek alternative treatments; the extent of acceptance of traditional medicines by the population in general; and the point of integration between western and traditional medicines in Cameroon.

Identification, classification and documentation of all major medicinal plants is also a concern as well as how to identify what has been lost from untransferred knowledge, particularly at the periphery. Additional concerns that should be addressed include whether traditional knowledge systems relating to sustainable use and conservation of medicinal plants are clearly understood; whether gender issues have been adequately considered; whether traditional methods can be incorporated into research strategies; how local and traditional knowledge may contribute to the propagation and sustainable use and conservation of medicinal plants; the limitations and potentials of spiritual beliefs relating to traditional healing systems; the cultural practices and beliefs that are supportive of the conservation and sustainable utilization of medicinal plants; as well as the benefits derived. A coherent research agenda for the Cameroon ethno-scientist should focus on: i) safety and efficacy of traditional remedies; ii) designation of appropriate research procedures and standardization of herbal medicines that are illegally inundating the market; iii) provide reasons for the utilization of traditional medicines extrapolating results to the area of medical, social, demographics and economics sciences; iv) defining the consumers of traditional medicines and stating conditions under which traditional medicines are most appropriate; v) conducting ethno-botanical surveys such as those of Jiofack *et al* (61,62) and the Red Data Book regarding the status of Cameroonian medicinal plants; vi) initiation of studies of indigenous conservation with a focus on the rapid disappearance of the natural flora used as medicinal plants; vii) effective agro-ecological methods that enhance the propagation and cultivation of specific medicinal plants with a particular focus on the encroaching desertification.

## 17. Conclusion

Cancer in particular and research in general is given very little attention in Cameroon. Attention is usually paid to infectious- and poverty-related diseases either due to their volatile and extremely sensitive nature or due to the lack of funding. Consequently, victims of cancer and other chronic diseases generate their own interventions to cope with their conditions, a ‘coping mechanism’. In most developing countries cancer has not been a priority for health ministries as nutritional, parasitic and infectious diseases have presented a greater and more immediate challenge. The level of interest in and development of cancer statistics may reflect the low priority that has been given to malignant disease in the past. There is the need in Cameroon for the continuous promotion of health research in cancer management and development of new chemical entities from the plethora of natural medicinal flora. Thus, cancer study awareness could be elevated to priority status (72,73).

## Figures and Tables

**Table I:** Cancer prevalence and mortality in Cameroon (73).

Characteristics	Male	Female	Both genders
Population (thousands)	9,538	9,549	19,088
Number of new cancer cases (thousands)	5.4	6.3	11.7
Age-standardized rate (W)	88.8	96.5	92.1
Cancer risk prior to age 75 (%)	8.2	9.2	8.7
Number of cancer deaths (thousands)	4.6	4.4	9.0
Age-standardized rate (W)	76.8	70.5	73.1
